# Equine Influenza: Epidemiology, Pathogenesis, and Strategies for Prevention and Control

**DOI:** 10.3390/v17030302

**Published:** 2025-02-21

**Authors:** Francesco Branda, Dong Keon Yon, Mattia Albanese, Erica Binetti, Marta Giovanetti, Alessandra Ciccozzi, Massimo Ciccozzi, Fabio Scarpa, Giancarlo Ceccarelli

**Affiliations:** 1 Unit of Medical Statistics and Molecular Epidemiology, Università Campus Bio-Medico di Roma, 00128 Rome, Italy; m.ciccozzi@unicampus.it; 2 Center for Digital Health, Medical Science Research Institute, Kyung Hee University Medical Center, Kyung Hee University College of Medicine, Seoul 02447, Republic of Korea; yonkkang@gmail.com; 3 Department of Regulatory Science, Kyung Hee University, Seoul 02447, Republic of Korea; 4 Department of Pediatrics, Kyung Hee University College of Medicine, 23 Kyungheedae-ro, Dongdaemun-gu, Seoul 02447, Republic of Korea; 5 Department of Public Health and Infectious Diseases, University of Rome Sapienza, 00161 Rome, Italy; dott.albanese.mattia@gmail.com (M.A.); erica.binetti@uniroma1.it (E.B.); 6 Hospital of Tropical Diseases, Mahidol University, Bangkok 10400, Thailand; 7 Sciences and Technologies for Sustainable Development and One Health, Università Campus Bio-Medico di Roma, 00128 Rome, Italy; giovanetti.marta@gmail.com; 8 Climate Amplified Diseases and Epidemics (CLIMADE), Belo Horizonte 30190-002, MG, Brazil; 9 Instituto Rene Rachou, Fundação Oswaldo Cruz, Belo Horizonte 30190-009, MG, Brazil; 10 Department of Biomedical Sciences, University of Sassari, 07100 Sassari, Italy; aciccozzi@uniss.it (A.C.); fscarpa@uniss.it (F.S.); 11 Azienda Ospedaliero Universitaria Umberto I, 00185 Rome, Italy; 12 Migrant and Global Health Research Organization—Mi-Hero, 00185 Rome, Italy

**Keywords:** equine influenza, H3N8 subtype, epidemiology of equine diseases, cross-species transmission, vaccination strategies, biosecurity measures, genetic surveillance, one health

## Abstract

Equine influenza (EI) is a highly contagious respiratory disease caused by the equine influenza virus (EIV), posing a significant threat to equine populations worldwide. EIV exhibits considerable antigenic variability due to its segmented genome, complicating long-term disease control efforts. Although infections are rarely fatal, EIV’s high transmissibility results in widespread outbreaks, leading to substantial morbidity and considerable economic impacts on veterinary care, quarantine, and equestrian activities. The H3N8 subtype has undergone significant antigenic evolution, resulting in the emergence of distinct lineages, including Eurasian and American, with the Florida sublineage being particularly prevalent. Continuous genetic surveillance and regular updates to vaccine formulations are necessary to address antigenic drift and maintain vaccination efficacy. Additionally, rare cross-species transmissions have raised concerns regarding the zoonotic potential of EIV. This review provides a comprehensive overview of the epidemiology, pathogenesis, and prevention of EI, emphasizing vaccination strategies and addressing the socio-economic consequences of the disease in regions where the equine industry is vital.

## 1. Introduction

Equine influenza (EI) is a highly contagious and acute respiratory disease that continues to pose a significant threat to equine populations worldwide [1]. The causative agent, Equine influenza virus (EIV), is an enveloped, single-stranded, negative-sense RNA virus belonging to the *Alphainfluenzavirus* genus of the *Orthomyxoviridae* family [2,3] and is classified as an influenza A virus [4]. Its eight-segmented genome allows for frequent genetic reassortment, contributing to its antigenic variability and complicating efforts for long-term disease control [5]. Although EIV infection is rarely fatal, its high transmissibility via aerosolized respiratory secretions, direct contact, and fomites results in widespread outbreaks, leading to significant morbidity among susceptible equine populations [6]. The economic consequences of these outbreaks are profound, encompassing costs associated with veterinary care, quarantine measures, and disruptions to equestrian sports, breeding programs, and recreational activities [1]. These impacts underscore the critical need for effective preventive measures, particularly vaccination [7]. EIV is primarily associated with two subtypes of influenza A viruses, H7N7 and H3N8, distinguished by antigenic differences in their surface glycoproteins hemagglutinin (HA) and neuraminidase (NA) [8]. The H7N7 subtype, first identified in 1956 [9], was responsible for multiple outbreaks during the mid-20th century but has not been detected in equine populations since the late 1970s. Its disappearance remains poorly understood, with theories ranging from an evolutionary disadvantage to competitive exclusion by the H3N8 subtype [10,11]. Conversely, the H3N8 subtype, first isolated in 1963 [12], circulates globally and is the predominant cause of EI [13,14,15,16]. The H3N8 EIV has undergone substantial antigenic evolution, leading to the emergence of two distinct lineages: the Eurasian and the American [17,18,19]. The American lineage has further diverged into Argentinian, Kentucky, and Florida sublineages [20]. Of these, the Florida sublineage dominates, with its Clade 1 circulating primarily in the Americas and its Clade 2 prevalent in Europe, Asia, and the Middle East [18,19]. This rapid evolution necessitates continuous genetic surveillance to ensure that vaccines remain effective against currently circulating strains. Although vaccination remains the cornerstone of EI prevention, antigenic drift and occasional antigenic shifts in the virus necessitate regular updates to vaccine formulations to maintain efficacy [21,22,23]. Additionally, the potential for cross-species transmission, as evidenced by rare instances of EIV infection in other mammal populations, raises concerns about the zoonotic potential of the virus and its broader epidemiological implications [23].

This review aims to provide a comprehensive synthesis of current knowledge on the epidemiology, pathogenesis, and prevention of EI, with a particular focus on vaccination strategies and their limitations. Additionally, it addresses the socio-economic consequences of the disease, particularly in regions where the equine industry constitutes a critical economic and cultural resource. By consolidating recent advancements, this review seeks to support the development of improved strategies for controlling and preventing EI, ultimately minimizing its global burden.

## 2. Global Epidemiology and Surveillance of Equine Influenza

### 2.1. Surveillance and Disease Reporting Systems in Equine Health: Initiatives and Platforms

The management and control of infectious diseases as they arise are critical to reducing the number of affected individuals within a population and limiting the economic and operational disruptions these diseases can cause. A timely and effective response can halt the wider spread of infections, reducing the risk of widespread outbreaks or even pandemics. To achieve the highest levels of control and prevention, infectious diseases are systematically monitored through national and international surveillance systems. There are two main ways that equine surveillance is conducted:

1. Mandatory reporting of notifiable diseases

Some equine diseases are classified as notifiable under veterinary or human health legislation, e.g., EI, African Horse Sickness (AHS), Contagious Equine Metritis (CEM), dourine, Equine Viral Arteritis (EVA), Equine Infectious Anemia (EIA), glanders, and West Nile Fever. These diseases are designated as reportable because of their potential impact on human and animal health as well as international trade. Reporting is mandatory when clinical signs raise a suspicion or the diagnosis is confirmed by testing, such as those recommended by the Horserace Betting Levy Board (HBLB) International Codes of Practice for Breeding Procedures. Response measures are tailored to the specific disease and national regulations, but often involve movement restrictions and testing of potentially exposed populations. Data collected during these surveys are analyzed and disseminated through platforms such as the World Animal Health Organization’s World Animal Health Information System (WOAH-WAHIS).

2. Voluntary disease investigation and reporting of positive laboratory test results for nonreportable diseases

For diseases not classified as reportable, diagnosis through laboratory tests may still be voluntarily reported by veterinarians or laboratories as part of a broader surveillance effort. Such reports are often accompanied by epidemiological data, including age, breed, vaccination history, geographic location, and recent movements of the horse. Additional pathogen analyses can be conducted to identify characteristics such as the strain involved, which help inform vaccination strategies and disease management. However, this voluntary system can lead to under-reporting, as the steps required to report cases depend on individual decisions. In addition, the types of cases reported may be skewed: the most severe cases or those from populations undergoing mandatory or subsidized testing are more likely to be documented. This can result in an incomplete picture of the true prevalence of the disease in a region or country.

**Figure 1:**
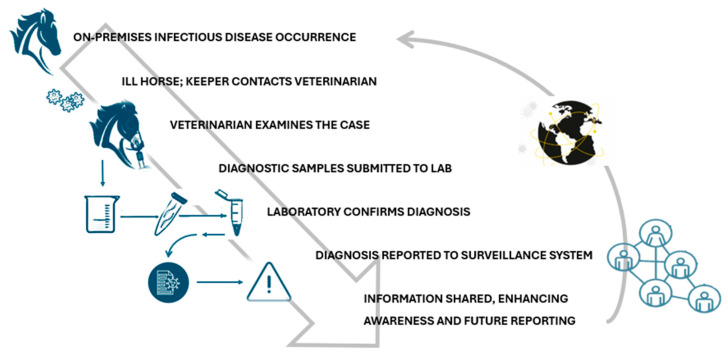
The pathway of surveillance (https://trainermagazine.com/european-trainer-articles/equine-infectious-disease-surveillance-in-northern-europe/2022/3/30, accessed on 18 December 2024).

Figure 1 provides a detailed overview of the equine infectious disease surveillance process, highlighting the critical steps involved in identifying and managing disease in equine populations. Below, each step is explained in more detail to illustrate the interconnected efforts that contribute to effective surveillance:

1. Detection of diseases on the farm: The initial phase begins with the recognition by an owner, janitor, or facility manager of signs of illness in one or more horses. These signs may include respiratory distress, fever, lethargy, or other clinical symptoms indicative of an infectious disease. In some cases, experience with previous outbreaks may allow early recognition, while less obvious signs may delay the identification process. This underscores the importance of education and awareness programs for equine facility workers to improve their ability to detect potential health threats early.
2. Contacting a veterinarian: Once a suspected disease is identified, the horse keeper promptly contacts a veterinarian. This step is critical, as veterinary expertise is needed to confirm whether symptoms correspond to an infectious disease and to initiate appropriate diagnostic procedures. Early communication with a veterinarian can also prevent the spread of disease by promptly activating isolation measures or movement restrictions within the facility.
3. Veterinary examination: At the time of the facility visit, the veterinarian performs a thorough examination of the affected horse. This may include observation of clinical signs, history, and evaluation of environmental factors such as proximity of other horses or recent movements of the horse. Based on these findings, the veterinarian collects diagnostic samples, such as nasal swabs, blood, or other biological materials. These samples are critical in determining the presence of specific pathogens, allowing for an accurate diagnosis.
4. Sending samples to the laboratory: The collected specimens are sent to a diagnostic laboratory specializing in veterinary infectious diseases. Ensuring proper storage and transport of these samples is essential to maintain their integrity and accuracy. The laboratory plays a central role in the surveillance process, employing advanced diagnostic methods, including PCR (polymerase chain reaction), serology, and culture techniques, to identify pathogens associated with equine diseases.
5. Laboratory diagnosis: In the laboratory, the submitted samples undergo detailed analysis to determine the causative agent of the disease. The results of these tests can confirm the presence of specific pathogens, such as equine influenza virus, equine herpesvirus, or bacterial agents such as *Streptococcus equi* (causative agent of strangles). Laboratories can also perform genetic or antigenic characterization of the identified pathogens to understand strain variations, which can provide insights for vaccine updating or treatment adjustment.
6. Reporting diagnosis: Once the laboratory confirms a diagnosis, the results are communicated to relevant surveillance programs or initiatives. This information is critical for tracking disease spread regionally, nationally, or even internationally. Reporting systems vary from region to region, but typically involve government authorities, industry organizations, and disease monitoring platforms. For example, data from confirmed cases may feed into databases maintained by organizations such as the World Organization for Animal Health (WOAH).
7. Sharing of anonymized data: The final stage of the surveillance process involves sharing anonymized data with stakeholders through disease reporting platforms. These data include information on the geographic location of outbreaks, observed clinical symptoms, and characteristics of the affected population, such as age, breed, and vaccination history. Anonymization ensures privacy and encourages greater participation of horse owners and facilities in future reporting.

Many countries have established programs to encourage diagnosis of equine infectious diseases, often providing financial incentives. In the United Kingdom, for example, equine veterinarians can send nasopharyngeal swab samples from horses showing signs of potential equine influenza to undergo free RT-PCR testing at licensed laboratories. This program, funded by the HBLB and managed by the Equine Infectious Disease Surveillance (EIDS) at the University of Cambridge, also supports a network of commercial laboratories that report positive cases and share epidemiological and virological data. These initiatives are critical to tracking equine influenza, given the virus’ ability to evolve into new strains that are potentially more infectious or resistant to existing vaccines. In addition to equine influenza, the UK actively tracks laboratory-confirmed cases of equine herpesvirus-1 (EHV-1), a pathogen capable of causing neurological problems, abortions, and foal mortality. Surveillance also extends to the highly contagious disease of strangles, with data collected to improve understanding and strategies for prevention and control. Internationally, several countries have developed platforms for notifying stakeholders of laboratory-confirmed cases of equine diseases. European examples include the French RESPE, Dutch SEIN, Belgian EFPB, and Swiss Equinella. Complementing these national efforts, the International Collating Centre (ICC), coordinated by EIDS, and supported by the International Thoroughbred Breeders’ Federation (ITBF) and Fédération Équestre Internationale (FEI), has been compiling outbreak reports for more than 30 years. The reports come from national contacts, veterinarians, and diagnostic laboratories and are disseminated to subscribers via e-mail alerts and quarterly summaries, archived on the ICC website. In 2019, ICC introduced an interactive online platform for users to analyze outbreak data, with records available from that year. This tool strengthens global communication on equine diseases, ensuring that stakeholders remain up-to-date. In addition, EIDS operates EquiFluNet, a dedicated platform for equine influenza surveillance in the UK and around the world, further enhancing international coordination in infectious disease management.

### 2.2. A Summary of the Recent Findings of EquiFluNet Surveillance Initiative

**Figure 2:**
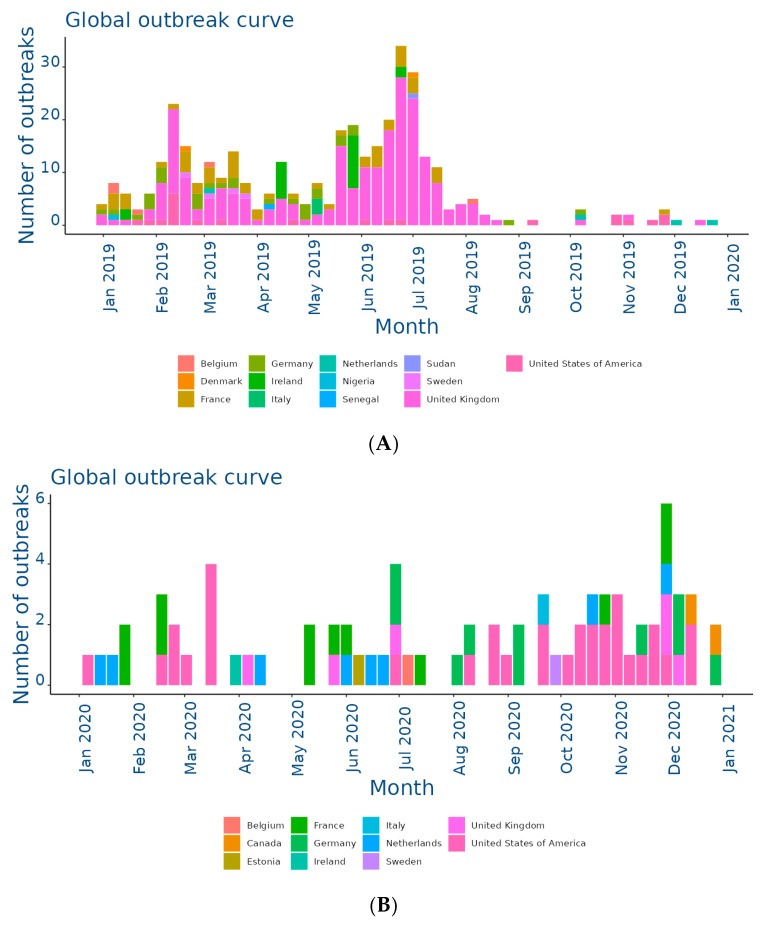

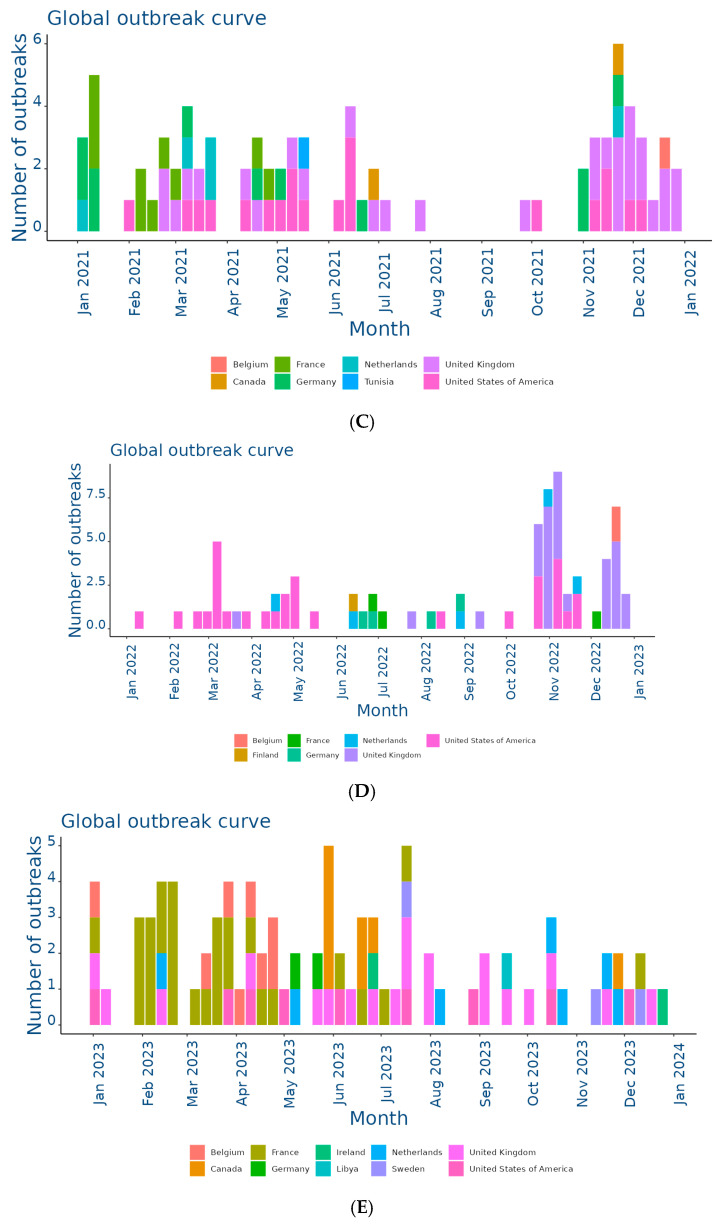

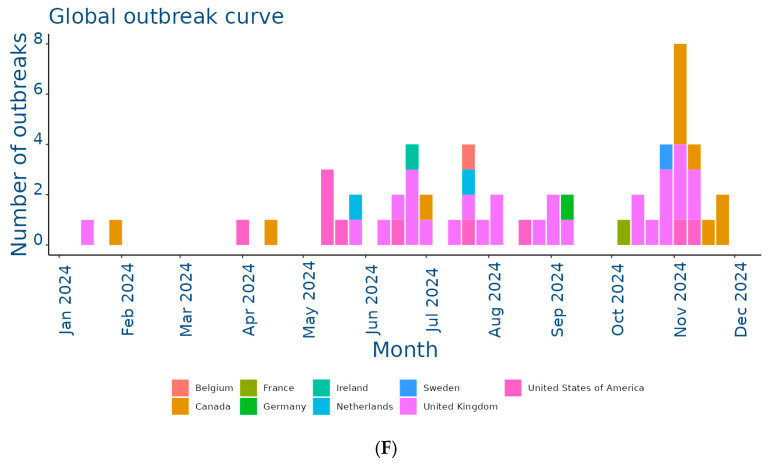
Outbreaks occur by month between (**A**) Jan and 31 Dec 2019; (**B**) Jan and 31 Dec 2020; (**C**) Jan and 31 Dec 2021; (**D**) Jan and 31 Dec 2022; (**E**) Jan and 31 Dec 2023; (**F**) Jan and 25 Nov 2024. Data and figures obtained from EquiFluNet (https://equinesurveillance.org/equiflunet/, accessed on 18 December 2024).

The EquiFluNet surveillance initiative, which monitored equine influenza outbreaks globally, revealed notable trends and fluctuations in the prevalence of the disease from 2019 to 2024. Data collected, as of 25 November 2024, highlighted significant geographic and temporal variability in outbreak numbers. Figure 2A showed an exceptionally high level of activity in 2019, with a peak in the UK, which recorded 229 outbreaks distributed throughout the year. This represented the highest value reported in all years considered. France and Germany contributed significantly to the total number, with 52 and 29 outbreaks, respectively. In 2019, outbreaks were concentrated in the winter and spring months, with a reduction during the summer months, indicating a seasonal pattern. Countries such as Belgium, the Netherlands, and Ireland showed moderate activity, while Italy, Finland, and other countries recorded negligible numbers.

In 2020, as shown in Figure 2B, there was a dramatic decline in outbreaks on a global scale. The UK, despite being the country with the most outbreaks in 2019, recorded only six cases in 2020. The US emerged as the country with the most activity, with 33 outbreaks evenly distributed throughout the year, while in Europe, numbers remained low, with France and Germany recording 12 and 10 outbreaks, respectively. This decline could be attributed, at least in part, to the containment measures put in place in response to the COVID-19 pandemic, which may have limited virus transmission among equines.

Figure 2C reflected relatively stable activity in 2021, although reduced compared to 2019. The main contributors were again the United Kingdom, with 30 outbreaks, and the United States, with 20 outbreaks. France and the Netherlands showed moderate activity, while countries such as Belgium and Ireland had a small but persistent number of outbreaks. The monthly trend showed a more even distribution than in previous years, suggesting a lesser impact of seasonality.

In 2022, the situation remained relatively stable, as illustrated in Figure 2D. The United States and the United Kingdom both reported 30 outbreaks, followed by countries with lower levels of activity, such as Germany and the Netherlands. However, there was a further reduction this year in countries with historically low prevalence, such as Italy, Denmark, and Finland, which reported no outbreaks. This reduction could have been due to improved control measures or reduced surveillance in some areas.

Figure 2E showed a significant increase in activity in 2023. In particular, France experienced a peak of 28 outbreaks, most of them concentrated in the spring and autumn months. Belgium and Sweden also showed a sporadic increase, with eight and three outbreaks, respectively. The United States and the United Kingdom, although showing a decrease compared to previous years, remained among the most affected countries. In Africa, a single outbreak was reported in Libya, highlighting the need for improved surveillance in this region.

Finally, Figure 2F provided data updated to 25 November 2024, showing a further reduction in the total number of outbreaks compared to 2023. The United Kingdom recorded 28 outbreaks, while the United States reported 10. Even Canada, which had recorded a small number of outbreaks in previous years, showed an increase with 11 cases. France, Germany, and the Netherlands reported negligible numbers, with only one case each. The monthly trend suggested a general decline in transmission, but continued monitoring was necessary to assess the risk of a resurgence.

Overall, although horse flu significantly affected countries such as the UK and the US, the situation in other areas was more variable, as summarized in Table 1. The UK emerged as the country with the highest total number of outbreaks, totaling 229 cases in 2019, significantly more than any other country. After a sharp drop in 2020 (six cases), numbers gradually increased to 28 outbreaks in 2024, demonstrating the resilience of the virus despite control measures. This trend reflected the importance of the UK as an international hub for equestrian competitions and horse trade, which could facilitate transmission. In the US, numbers ranged from a peak of 33 outbreaks in 2020 to a low of nine in 2023. The temporal distribution suggested that 2020 was a critical year for the outbreak, likely due to increased equine mobility prior to the adoption of pandemic restrictions. However, with 10 outbreaks in 2024, activity remained relatively low. France presented an interesting trend, going from 52 outbreaks in 2019 to only one case in 2024. The peak in 2023, with 28 outbreaks, represented a resurgence that interrupted a phase of steady decline. This could have been related to local events or increased surveillance. Similarly, Germany showed a declining trend from 29 cases in 2019 to a single outbreak in 2024, with intermediate variations reflecting reduced transmission. In the Netherlands, the number of outbreaks was more stable than elsewhere, varying between two and eight cases per year. This suggested a moderate endemic situation, likely effectively managed through vaccination and surveillance programs.

Several countries, including Belgium, Sweden, and Ireland, reported sporadic activity. Belgium, for example, went from five cases in 2019 to a peak of eight in 2023, before dropping to a single outbreak in 2024. Sweden and Ireland followed a similar pattern, with sporadic prevalence and overall low numbers. In Denmark and Finland, no outbreaks were recorded from 2022 onwards. This could have been due to climatic factors less favorable to virus transmission or the effectiveness of prevention strategies. In Africa and parts of the Middle East, reported activity was minimal. For instance, Libya recorded a single outbreak in 2023, while Nigeria, Senegal, and Sudan reported isolated cases in the early years of the study period. These data underlined the need to improve surveillance systems in these regions, where virus transmission might have been underestimated.

### 2.3. Modes of Transmission and Risk Factors for EIV Spread

**Figure 3:**
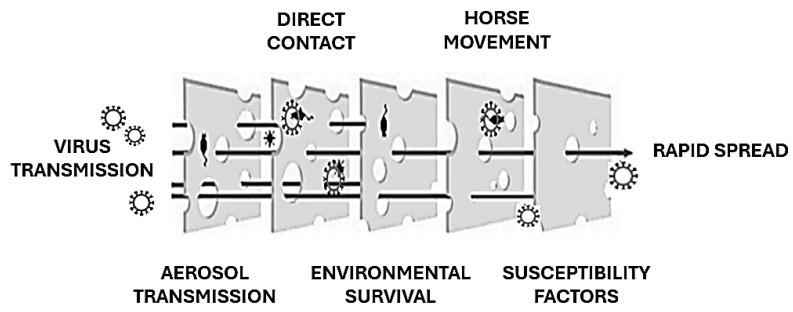
EIV spread explained using the “Swiss Cheese model” (adapted from [28]). Each preventive barrier against EIV transmission may have gaps.

The EIV is primarily transmitted through airborne respiratory droplets, which can remain suspended in the air and spread up to 1–2 km, contributing to rapid dissemination over large areas [6,24]. Transmission also occurs through direct contact with nasal secretions and indirectly via contaminated objects such as feed containers, grooming equipment, and tack. The virus’s capacity for swift transmission within equine populations often exceeds that of other respiratory pathogens affecting horses [6]. The global movement of horses, whether for trade, competition, or breeding purposes, plays a pivotal role in introducing EIV to regions where it was previously absent. Additionally, the virus can remain viable on surfaces for up to three days, allowing indirect transmission through contaminated materials [25]. The incubation period is brief, typically 1–3 days, with infected horses shedding significant amounts of virus in nasal secretions for up to 10 days, often before visible symptoms appear. Crowded conditions in stables and inadequate ventilation exacerbate the spread of the virus by increasing the likelihood of exposure among susceptible animals. EIV is highly infectious, with nearly all unvaccinated horses becoming infected during outbreaks. Young horses, particularly those between the ages of 1 and 5 years with limited or no prior exposure to the virus, are especially vulnerable. The immune status of individual horses heavily influences disease dynamics; animals with partial immunity from vaccination or prior exposure often exhibit subclinical infections, slowing the spread of the virus compared to fully susceptible individuals [26] (Figure 3). While EIV typically resolves without causing long-term infection, as the virus does not establish latency in recovered horses [27], its ability to cross species boundaries has raised significant concerns. For instance, the H3N8 strain of EIV was detected in dogs in Florida in 2004, likely due to close interaction with infected horses or the consumption of contaminated horse meat. This adaptability underscores the potential for EIV to exploit new hosts and ecological niches, as reported for other zoonotic viruses [28], posing broader epidemiological challenges [11].

## 3. Evolution and Pathogenicity of the Virus

### 3.1. Origin and Adaptations: Key Mutations and Persistence of H3N8 Worldwide

**Figure 4:**
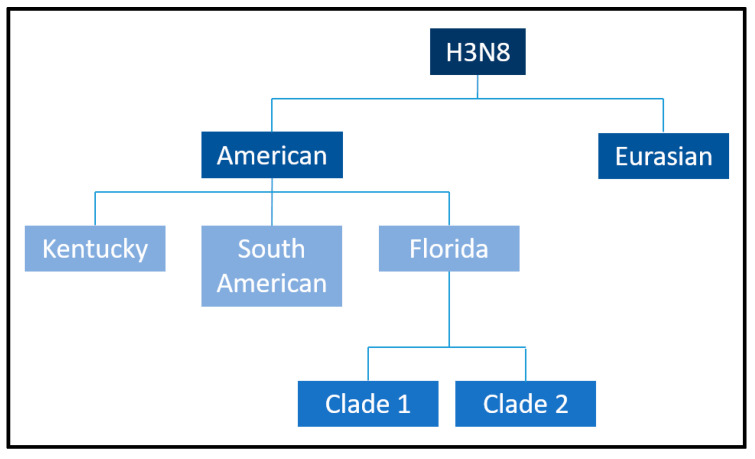
Equine influenza H3N8 American and Eurasian sublineages. Borrowed from: Equine influenza (flu). Amy Young, 8 July 2019. UCDAVIS. Veterinary medicine (Center for Equine Health). Available online: https://ceh.vetmed.ucdavis.edu/health-topics/equine-influenza-flu, accessed on 18 December 2024. The image has been edited by using the software GIMP 2.0 available at https://www.gimp.org, accessed on 18 December 2024.

Initial phylogenetic analyses suggested that H3N8 evolved as a single lineage [29]. However, subsequent sequencing of the HA gene revealed its divergence into two primary genetic and antigenic lineages: the Eurasian and American lineages (Figure 4), a division that likely occurred in the 1980s [17]. Within the American lineage, further diversification gave rise to the Argentinian, Kentucky, and Florida sublineages [20]. The Florida sublineage has since split into two clades, termed Florida Clade 1 (FC1) and Clade 2 (FC2), characterized by distinct HA sequences [30]. FC1 predominantly circulates in the Americas, while FC2 is responsible for most outbreaks in Europe, Asia, and other regions [18]. However, both clades have demonstrated the ability to spread across geographic barriers, as evidenced by FC1 outbreaks in Japan, Australia (2007–2008), and Europe [14,31,32] and FC2 outbreaks in China, India, and Mongolia [33]. Molecular surveillance has revealed additional mutations, such as A144V and I179V in FC2 viruses, which affect antigenic regions but do not impact the results of HI assays using ferret antisera [22,34]. The I194V mutation has also been identified as a consistent marker in all American lineages, distinguishing them from their Eurasian counterparts.

The global persistence of H3N8 EIV is closely associated with its capacity for antigenic evolution and the acquisition of adaptive mutations that enhance viral fitness and transmission [11]. Several studies have reported that key amino acid substitutions in the HA protein play a pivotal role in modulating antigenicity and facilitating immune evasion strategies [35]. Specifically, changes at residues 159, 189, and 227 are critical for altering antigenicity, as shown in studies using reverse genetics and hemagglutination inhibition assays [35]. Residue K189 within antigenic site B plays a pivotal role in determining the antigenic profile of the virus and distinguishing between Eurasian and American sublineages [17,36]. Moreover, the substitution of uncharged, acidic, or basic amino acids in this position significantly affects the antigenic properties and contributes to immune evasion [37]. Notably, similar mutations in human H3N2 viruses have been implicated in vaccine failures, such as during the 2005–2006 outbreak in Iran [38,39], underscoring the relevance of HA mutations in shaping viral persistence.

In a recent study, Kleij and colleagues [15] conducted a genetic analysis of H3N8 EIV isolates from France, focusing on those circulating during the winters of 2009 and 2018, aiming to identify key virulence factors and assess antigenic properties of the virus. A comparison of the HA sequences between French EIV strains and the OIE-recommended A/equine/Ohio/1/2003 (FC1) strain identified twenty-two amino acid substitutions in an H3N8 equine strain isolated in 2018. Among these, five substitutions (A138S, T163I, N188T, R62K, and N63D) were found in key antigenic sites: site A for A138S, site B for T163I and N188T, and site E for R62K and N63D. The presence of these substitutions within the antibody-binding regions of HA may contribute to antigenic drift, potentially altering the virus’s immune evasion ability [15]. Among circulating FC2 EIV strains and the OIE-recommended vaccine strain A/equine/Richmond/2007 (FC2), only four substitutions were identified at antigenic sites [15]. Wilson and Cox [40] suggested that four or five amino acid changes in two distinct antigenic sites could enable a virus to escape preexisting immunity, potentially resulting in vaccine failure for human influenza A viruses. In the case of equine influenza A viruses, a difference of 10 to 16 amino acids between the circulating outbreak strains and the vaccine strains may be sufficient to cause a breakdown in vaccine effectiveness [35,41]. These results have confirmed that current vaccines appear to remain effective against both FC1 and FC2 strains.

Equine-specific genetic markers in the polymerase basic protein 1 (PB1), polymerase acidic protein (PA), and PB2 proteins, associated with adaptation from avian to equine hosts, remained largely conserved in recent strains [15]. However, certain reversion mutations, such as PA E237 reverting to the avian K237 in recent FC1 and FC2 strains, could evidence ongoing evolution [15]. Substitutions at PB2 positions 661 and 684, known to facilitate mammalian adaptation, were also identified in recent strains, underscoring their potential role in viral fitness [15]. Finally, the accessory protein PB1-F2, implicated in modulating pathogenicity, varies significantly between strains. Notably, a full-length PB1-F2 protein (90 amino acids) in the A/equine/Paris/1/2018 strain, with enhanced mitochondrial membrane permeabilization compared to the shorter (81 amino acids) PB1-F2 in A/equine/Beuvron-en-Auge/2/2009, may influence virulence, though further studies are needed to confirm its role in equines [15].

### 3.2. Cross-Species Transmission of H3N8 and Host Range Expansion

EIV’s ability to infect non-equine hosts highlights its potential for cross-species transmission. The canine influenza virus (CIV), H3N8 subtype (A/canine/Colorado/30604/2006), originated from EIV but acquired mutations enabling it to bind to receptors in the canine respiratory tract [42]. Despite these changes, CIV remains minimally pathogenic to horses and does not establish sustained infections in equids [26,43]. Conversely, transmission of EIV to dogs has been documented, particularly in the UK and Australia, indicating a potential bidirectional transmission between species [44]. These viruses show minimal differences in their genetic makeup, including mutations in the PA-X protein, which may play a role in host adaptation [45]. Experimental studies have further confirmed interspecies transmission potential. Horses infected with EIV have been shown to transmit the virus to co-housed dogs, leading to viral shedding and seroconversion, although clinical signs in dogs were absent [46]. Conversely, when CIV-infected dogs were housed with uninfected horses, no evidence of transmission, viral shedding, or seroconversion was observed, underscoring the species–specific barriers to sustained cross-transmission [47]. EIV has also been sporadically detected in other species. For instance, an H3N8 strain was isolated from a Bactrian camel in Mongolia in 2012, marking a rare spillover event [48]. Experimental studies have shown that cats can also be infected with EIV under controlled conditions, demonstrating the virus’s broader host range [49]. The presence of EIV in pigs raises concerns about its potential role in genetic reassortment. In China, H3N8 EIV strains were isolated from swine, highlighting the potential for cross-species infection [50]. Pigs, possessing both α-2,3 and α-2,6-linked sialic acid receptors, are considered “mixing vessels” for influenza viruses, capable of facilitating genetic reassortment. Although EIV infections in pigs do not typically result in clinical signs or severe pathology, the potential for reassortment remains a concern [51]. Phylogenetic analyses indicate that equine and canine H3N8 viruses have distinct evolutionary trajectories, with limited transmission between the two species [52]. Additionally, experimental infections with H3N8 strains from other hosts, such as seals and birds, suggest that these viruses can replicate in pigs and cause detectable lesions, further underscoring their pathogenic potential [53]. Given these findings, continuous surveillance and genetic characterization of H3N8 across different species are essential to understand its transmission dynamics, minimize spillover events, and mitigate the risk of reassortment leading to novel zoonotic strains.

### 3.3. Pathogenic Mechanisms: Viral Interactions and Clinical Manifestations in Horses

The EIV primarily targets the ciliated epithelial cells of the upper and lower respiratory tract, impairing the clearance of foreign particles and facilitating infection [8]. The HA glycoprotein on the viral surface binds to sialic acid receptors on the host cell membrane, initiating receptor-mediated endocytosis. The virus is subsequently internalized into an endosome, where the acidic environment triggers the viral envelope and the endosomal membrane fusion due to a conformational change of HA. Low pH also activates the M2 ion channel, acidifying and so facilitating the release of the viral genome, which is translocated to the host nucleus. The viral genome consists of eight segments (coding one or more viral proteins) forming eight ribonucleoprotein complexes (RNPs), each of them includes one RNA segment, a nucleoprotein (NP), and three proteins (PA, PB1, and PB2) of a viral RNA-dependent RNA polymerase (RdRp) [54]. The viral RNA-dependent RNA polymerase (RdRp) then drives RNA synthesis, hijacking the host cellular machinery to produce viral components. Once viral protein synthesis is complete, vRNPs exit the nucleus, and new viral particles assemble and bud from the host cell [8]. Viral replication results in cell-to-cell spread within the respiratory epithelium, culminating in epithelial necrosis, protein-rich fluid exudation into the airways, ciliary dysfunction, and impaired mucociliary clearance. The equine respiratory epithelium is rich in Neu5Gc2-3Gal moieties, which are critical for EIV replication, with the viral NA exhibiting a high affinity for these receptors. This interaction facilitates progeny virus release during early infection [55,56]. Infected cells often undergo apoptosis, a hallmark of EIV-induced cytotoxicity, driven by caspase activation and cleavage [57,58]. Non-structural protein 1 (NS1) plays a pivotal role in pathogenesis by promoting viral replication and inhibiting host antiviral defenses, including RNA processing and immune signaling pathways such as interferon regulatory factor 3 (IRF-3) and NF-κB [59,60]. Cytotoxic T-cell responses targeting viral proteins such as M, NP, and PB2 are essential for viral clearance [26]. Additionally, host microRNAs (miRNAs) theoretically possess antiviral potential, but influenza A viruses, including EIV, may have evolved mechanisms to evade miRNA-mediated inhibition [61]. EIV shedding typically lasts 7–10 days, with viral RNA detectable by PCR for 15 days or longer [8]. While infection is rarely fatal in horses, colostrum-deprived neonates are particularly vulnerable. Secondary bacterial infections, such as those caused by *Streptococcus equi* subspecies *zooepidemicus*, exacerbate inflammation and can lead to bronchopneumonia [8]. Recovery of the respiratory epithelium takes approximately three weeks, during which the risk of secondary bacterial invasion remains heightened [26,62]. Histopathological findings in EIV-infected lungs commonly include bronchiolitis with serous exudates, diffuse pulmonary consolidation, necrosis of bronchiolar and alveolar structures, neutrophilic infiltration, hyaline membrane formation, and epithelial hyperplasia with squamous metaplasia [63]. The nasal cavity of equines harbors a protective mucus layer and sialic acid receptors capable of masking influenza receptor sites, thereby reducing HA binding and limiting infection [64]. Notably, strains such as EIV-A/equine/South Africa/2003 have caused respiratory pathology in dogs that mirrors CIV infections, suggesting a potential for cross-species transmission and greater pathogenicity compared to older EIV strains [65]. Studies using murine models have demonstrated that the disease progression, lesion patterns, and viral recovery in these models closely mimic natural and experimental equine infections. This highlights their potential utility for pre-clinical evaluation of EIV vaccines [66,67].

## 4. Prevention and Control of Equine Influenza

Prevention and control of the equine influenza is a key pillar in the epidemic management and protection of global equine populations. Integrated strategies, combining biosecurity measures, vaccination, and monitoring are essential to mitigate the impact of the disease.

### 4.1. The Critical Role of Vaccination in Equine Influenza Control

Vaccination is the most effective preventive measure against equine influenza. Vaccines available for equine influenza are generally inactivated virus, and, more recently, vaccines based on new technologies, such as messenger RNA, have been developed. Studies have shown that the use of updated vaccines, which include the most recent strains of the virus, can significantly improve the effectiveness of protection against the disease. In particular, vaccines that protect against H3N8 FC1 and FC2 virus variants have shown significant efficacy in recent outbreaks, such as the one that occurred in the UK in 2019 [68]. However, the protection conferred by vaccines tends to diminish over time, which is why regular recalls are necessary to ensure an adequate level of immunity. Continued research into the creation of more durable vaccines and innovative platforms such as mRNA vaccines is promising. These vaccines could offer longer protection and a faster response to new virus variants [23]. Despite the proven efficacy of vaccines, limited access to vaccines, particularly in countries with limited resources, is a challenge. Many low-income countries are unable to access up-to-date vaccines, compromising the effectiveness of global vaccination campaigns. In addition, the lack of adequate health infrastructure makes it difficult to monitor and respond to outbreaks in a timely manner.

### 4.2. Biosecurity and Surveillance: Complementary Measures in Outbreak Control

Biosecurity measures are essential to limit virus transmission among equines. These include isolation of infected animals, movement control, and regular disinfection of equine facilities. The adoption of strict biosecurity protocols has been shown to significantly reduce the risk of outbreaks, as evidenced during the equine influenza outbreak in Great Britain in 2019, when timely measures limited the spread of the virus [68]. Control of horse movements, particularly during equestrian events and competitions, is crucial, as these events can facilitate large-scale virus transmission. Training and education of operators are essential to ensure that biosecurity measures are taken correctly and uniformly. In addition, equine facilities must adopt frequent sanitization protocols, both for the environments and the equipment used, such as saddles and transport cages. The effectiveness of these measures is amplified by their continuous and uniform application in all high-density horse facilities.

Surveillance is crucial to detect equine influenza outbreaks early and prevent a large-scale spread. The EquiFluNet initiative, which globally monitors equine influenza outbreaks, has provided essential data to track the spread of the disease and respond quickly. Genetic surveillance of the virus is another powerful tool to monitor virus evolution, identify new variants and adapt control strategies accordingly. This approach has made it possible to quickly detect strains causing outbreaks and to update vaccines accordingly. The use of advanced technologies such as genome sequencing has greatly improved the ability to respond to outbreaks, enabling detailed analysis of virus variants [69]. The use of advanced technologies, in particular whole genome sequencing (WGS), has significantly improved the investigation of epidemic outbreaks, providing detailed information on viral phylogenetics, transmission dynamics, and molecular markers associated with pathogenicity [70]. Genomic epidemiology has been widely used in both human and veterinary medicine, including for seasonal human influenza, highly pathogenic avian influenza (HPAI), and West Nile virus (WNV), facilitating real-time monitoring of viral evolution and informing public health interventions [71]. For instance, WNV WGS revealed patterns of viral introduction and dispersal in different ecological niches, aiding vector control strategies [72]. Applications similar to equine influenza could improve strain selection for vaccines and contribute to a better understanding of the risks of interspecies transmission [13]. In parallel, predictive technologies based on artificial intelligence (AI) and machine learning (ML) have emerged as powerful tools for outbreak prediction, risk assessment, and resource optimization. AI-based models have been successfully applied in the early detection of zoonotic outbreaks, as demonstrated by predictive frameworks for Ebola [73], Nipah virus [74], and SARS-CoV-2 [75]. In the context of avian influenza, AI has been used to model viral spread based on environmental, climatic, and migratory patterns, leading to improved surveillance strategies [76]. AI-assisted epidemiological models have also been used for human influenza prediction, integrating mobility data and climatic factors to predict seasonal epidemic peaks [77]. Although AI and genomic epidemiology are not yet fully integrated into routine equine influenza surveillance, their potential applications are significant. AI models could improve the ability to identify high-risk regions, optimize vaccination programs, and improve real-time decision-making during outbreaks [78]. Similarly, metagenomic sequencing and AI-driven variant analysis could refine molecular surveillance efforts, ensuring that equine influenza vaccines remain effective against evolving strains. A One Health approach incorporating these advanced methodologies could significantly strengthen preparedness and response strategies for equine influenza and other equine infectious diseases [79].

Because equine influenza is a disease that can spread rapidly globally, a coordinated response between countries is critical. International collaboration enables the sharing of resources, information, and best practices and is essential to ensure that responses are timely and effective. International organizations such as the WOAH and the Food and Agriculture Organization of the United Nations (FAO) play a crucial role in coordinating global efforts against equine influenza. Cooperation between developed and developing countries is particularly important, as many low-income regions lack adequate infrastructure to deal with outbreaks [69]. Global challenges, such as emerging pandemics and health crises, require continued collective efforts to improve access to vaccines, strengthen surveillance capacities, and ensure that all countries have the necessary resources to respond to outbreaks in a timely manner. Despite progress, many challenges remain. Virus evolution remains a major concern, as the emergence of new variants could compromise the efficacy of existing vaccines. It is crucial that research continues to focus on improving vaccines, both in terms of duration of protection and ability to deal with emerging variants. Furthermore, the creation of universal and longer-lasting vaccines that require fewer recalls is a promising direction. Another area of research interest concerns sequencing and genetic monitoring technologies, which allow the spread and evolution of the virus to be tracked more precisely. This would enable a faster and more efficient response to epidemics. Promoting greater international cooperation in the research and distribution of vaccines is crucial to ensure that prevention measures are globally accessible, reducing the risk of large-scale outbreaks.

## 5. Potential Infectivity of the Equine Influenza Virus in Humans

While equine influenza is well-known in the veterinary field, its potential to infect humans is a subject of ongoing scientific inquiry. Influenza A viruses are known for their ability to cross species barriers. While human infections with equine influenza viruses are extremely rare and not well-documented, concerns remain regarding the virus’s potential for zoonotic transmission. The ability of influenza viruses to mutate or reassort genetic material raises the possibility that equine influenza viruses could adapt to humans, particularly in populations with prolonged exposure to infected horses, such as equine caretakers, veterinarians, or stable workers.

Few data in the literature, hypothesizing clinically evident infections caused by EIV in humans, are available.

The debate on the potential transmission of the infection to humans is still ongoing. Serological studies conducted in 1965 detected the presence of EIV antibodies in the elderly population, suggesting that EIV may have been responsible for the influenza pandemic of 1889. However, further studies are needed to confirm or refute this hypothesis [80]. In some countries, including Australia, serological evidence of the infection has been reported, particularly in individuals exposed to horses [81].

In the study conducted by Nyamdavaa Khurelbaatar et al. in Mongolia, 439 adults were enrolled in a prospective cohort study. They were followed during this period, and serological examinations were performed for various viruses. Thirty-seven participants had detectable antibody titers, 21 of which were against H3N8 [82]. However, the previously referenced studies employed classical serological techniques, including hemagglutination inhibition (HI) and virus neutralization tests (VNT). As such, there exists a potential bias that the detected antibodies may not have been generated specifically against EIV, as they could equally arise from infection with a non-equine H3 influenza virus. Finally, studies conducted on human volunteers have demonstrated subclinical or mildly symptomatic infections in approximately 66% of participants in whom the virus was inoculated [83]. To date, no confirmed human infection has been reported. A possible case of equine influenza infection in humans was described in Chile in 1973. A veterinary student, during a period of contact with horses infected with EIV, developed an influenza-like syndrome characterized by high fever, cough, rhinitis, difficulty breathing, and swelling of the submaxillary lymph nodes, which resolved within five days. Unfortunately, due to technical limitations, it was not possible to identify the virus [84]. In all these studies, virus detection, antigen detection, or serological testing were used to demonstrate the interaction between men and the virus. Due to the lack of concrete evidence of human cases, the primary purpose of implementing preventive measures currently remains to mitigate the spread of the virus within the horse population.

Monitoring of influenza viruses in horses remains critical to detect any potential changes that could increase the risk of zoonosis. Surveillance systems are essential to monitor equine influenza outbreaks and their evolution. Ongoing research focuses on understanding the virus’s genetic properties, host adaptability, and risks to public health. The WHO and veterinary organizations emphasize the importance of a “One Health” approach, which considers the interconnected health of humans, animals, and the environment. Although equine influenza has not been shown to infect humans on a large scale, its ability to infect multiple species and its evolutionary potential warrant continued vigilance. Close monitoring, research, and preventive strategies are vital to mitigating any future risks of zoonotic transmission.

## 6. Conclusions

Equine influenza remains one of the most relevant viral diseases for equine health globally, despite significant advances in disease prevention and control. Although management strategies, such as vaccination and biosecurity measures, have reduced the incidence of outbreaks in many regions, the disease remains a complex challenge, exacerbated by its highly contagious nature, geographical variability, and the continuing evolution of the virus. Data collected by global surveillance initiatives, such as EquiFluNet, show considerable variability in equine influenza outbreaks. Countries such as the United Kingdom and the United States experienced significant numbers of outbreaks, particularly in 2019 and 2020, but thereafter a decline in cases was observed, suggesting the effectiveness of the control measures taken. However, in many other regions, such as parts of Africa and Asia, outbreaks may remain underreported due to limited surveillance, which could hide a greater spread of the disease. In addition, the COVID-19 pandemic has had an impact on the dynamics of equine influenza spread, reducing animal mobility and the events that typically favor virus transmission. However, despite the decrease in cases in some countries, the virus continues to circulate at significant levels, as evidenced by the persistence of outbreaks in Europe and North America, which have seen an increase in cases in certain years. Vaccination remains the most effective prevention strategy to limit the spread of the virus and reduce the severity of the disease. However, the limited duration of protection conferred by vaccines requires regular recalls to maintain an adequate level of immunity, increasing the costs and resources required. In addition, the antigenic evolution of the virus requires frequent updates of vaccine formulations to address new emerging variants. Innovations in vaccines, such as messenger RNA vaccines, could offer more durable and adaptable solutions, but their large-scale availability requires significant investment.

Another major challenge is biosecurity measures, which are essential to limit the spread of the virus. Although measures such as isolation of infected animals and disinfection of facilities are widely adopted, their application can vary greatly depending on the resources available and the awareness of those responsible for equine facilities. Operator training and education on infectious disease management are critical to the success of biosecurity measures. Despite progress in global surveillance, implementation of monitoring systems remains insufficient in many areas, where lack of resources prevents timely data collection and dissemination. International collaboration is key to addressing this gap, promoting the sharing of information and resources to ensure a rapid and coordinated response to outbreaks. Finally, although considerable progress has been made in controlling equine influenza, the continuing evolution of the virus, limited access to vaccines in less developed regions, and the need for more affordable and accessible solutions remain major challenges. Addressing them will require continued efforts in research, technological innovation, and the promotion of closer international cooperation to ensure that all regions have access to effective prevention strategies. In this context, intensified surveillance, improved biosecurity measures, development of new vaccines, and expanded access to treatment are essential to reduce the impact of equine influenza on global equine health.

## Figures and Tables

**Table 1:** Countries reporting equine influenza outbreaks through EquiFluNet for 2019, 2020, 2021, 2022, 2023, and 2024 (as of 25 November).

Country	2019	2020	2021	2022	2023	2024
Belgium	5	1	1	2	8	1
Canada	0	2	2	0	8	11
Estonia	0	1	0	0	0	0
Denmark	2	0	0	0	0	0
Finland	0	0	0	1	0	0
France	52	12	10	3	28	1
Germany	29	10	11	4	2	1
Ireland	21	1	0	0	2	1
Italy	3	1	0	0	0	0
Libya	0	0	0	0	1	0
Netherlands	4	8	5	5	7	2
Nigeria	1	0	0	0	0	0
Senegal	1	0	0	0	0	0
Sudan	1	0	0	0	0	0
Sweden	4	1	0	0	3	1
Tunisia	0	0	1	0	0	0
United Kingdom	229	6	30	30	20	28
United States of America	22	33	20	31	9	10

## Data Availability

The data presented in this study are available at https://equinesurveillance.org/equiflunet/, accessed on 18 December 2024, and come from the International Collating Centre (ICC), operated by EIDS, which provides comprehensive surveillance reports on various equine infectious diseases.
